# Economic costs and Predictors of occupation-related Injuries in Ethiopian sugar industries from the Employer’s perspective: top-down approach and friction method

**DOI:** 10.1186/s12889-022-14519-5

**Published:** 2022-11-17

**Authors:** Mitiku Bonsa Debela, Muluken Azage, Negussie Deyessa, Achenef Motbainor Begosaw

**Affiliations:** 1 School of Public Health, College of Medicine and Health Sciences, Bahir Dar University, Bahir Dar, Ethiopia; 2 Department of Preventive Medicine, Schools of Public Health, College of Health Sciences, Addis-Ababa University, Addis-Ababa, Ethiopia

**Keywords:** Economic costs, The employers, Occupation-related injuries, Ethiopia

## Abstract

**Background:**

For many industrial workers, occupational injuries are a common health and safety concern. However, sufficient information on the economic costs and predictors of occupation-related injuries from the perspective of employers is lacking in developing countries, including Ethiopia. The objective of this study was to close this gap by quantifying the economic costs and predictors of occupation-related injuries in Ethiopian manufacturing industries from the employer’s perspective**.**

**Methods:**

A cross-sectional study was employed to estimate the employer-side economic cost of occupation-related injuries from December 2021 to March 2022. This study used a top-down approach to compute direct costs, while the friction method was used for indirect cost estimation. Injury data were obtained from the Bureau of Labour and Social Affairs and the industries, while cost data were from workers’ compensation records. The insurance company’s injury compensation record was triangulated with industries’ data. The study collected primary data via an interview-administered, semi-structured questionnaire from 1136 randomly selected injured cases. Statistical analysis was carried out with STATA version 14 software. The study employed a generalized linear model to identify predictors of total cost by considering the non-normal distribution of the total cost. Exponentiate coefficients with a 95% confidence interval were used to express the direction and strength of the association**.**

**Results:**

The survey participation rate was 100%. From the perspective of the employers, the total cost of occupation–related injury was 22,587,635.32 Ethiopian birr (537,800.84 $).Indirect and direct costs accounted for 65.86 and 34.14% of the overall expenses, respectively. Long-term absence from work (exp (b) = 0.85), having a sleeping disorder (exp (b) = 0.90), co-morbidity (exp (b) = 0.85), and severity (type) of injury (exp (b) = 1.11) were predictors significantly associated with the total cost variability in the fully adjusted model.

**Conclusions:**

Employers’ toll of occupation-related injuries has severe economic implications. The influential factors that elevated the total cost variation were: long-term absence from work, unsafe acts of the workers, having a sleeping disorder, co-morbidity, and severity (type) of injury. Therefore, the identified modifiable factors are the areas of intervention to reduce the cost of occupation-related injuries.

## Introduction

Occupational injury refers to a physical injury that a worker encounters while working that caused an illness resulting in death or work absences [1]. Globally, 2.78 million workers die from occupational accidents annually, while 374 million suffer from non-fatal occupational accidents [2, 3]. This occupation-related morbidity and mortality are becoming a substantial public health concern associated with high economic costs. Economic costs are borne by those injured workers, the government, and employers. The costs can be in the form of direct and indirect costs [4]. Despite successful declines in developed countries, which could be attributed to a decrease in occupational injury, reliable information on the current trends of the cost of work-related injuries are scarce in developing countries [5]. One study reported that the cost of occupational injuries was $3 trillion [6]. Evidence from international labor organizations showed that work-related injury’s financial costs range from 1.8 to 6% of Gross Domestic Product [7]. In America alone, the total cost of occupational injuries was $250 billion making the compensation cost $4 million per year [8], in Thailand $14 million [9], in the United Kingdom $14 billion [10], in Turkey, the cost of workday loss caused by major occupational accidents was 19,431.75$ [11]. Regarding risk factors, long working hours, shift work, job type, labor-intensive work, age over 65, and prior injury history increase the risk of occupational injury [12–15].

In Africa, the burdens of work-related injury are a more pervasive problem. The finding from Nigeria shows that the costs of occupational injury were $244,330,386 and $34,416.1 for fatal and severe injuries, respectively [16]. Another evidence from Kenya revealed that about 18.75% of employees missed 1–7 days or more than 1 month due to occupational injuries [17]. In Ethiopia, the prevalence of occupational injuries is a widespread problem [18–20]. Besides, several studies have documented high magnitude of occupation-related injuries in manufacturing industries [21–24]. However, sufficient information on the economic burdens and predictors of occupational-related injuries from the perspective of employers is still lacking. The objective of this study was to close this gap by quantifying the economic burden of occupational-related injuries and their predictors from the perspective of the employers in Ethiopia’s manufacturing industries. The employer has legal liability or is obligated to pay compensation for occupational injuries sustained at work. Also, basic benefits such as health insurance and sick leave are standard for employers of choice in our study. This study provides the scientific and policymaking communities with the information they need to intervene in the problem.

## Methods

### Setting, design and time –dimension

A cross-sectional study design was employed in Ethiopia’s two largest manufacturing industries (Metehara and Wonji sugar industries). Data extraction was done from December 14th, 2021, to March 23rd, 2022. The study used a retrospective costing approach to estimate the employer’s level of the economic burden of occupational injury. In this study, the problem was approached via an analysis of prevalence-based analysis that focuses solely on the costs incurred in a specific year, irrespective of the injury’s date.

### Inclusion criteria

We included any injured workers with lost workday during 2021. Besides, the study included any injured cases with insurance compensation payment through workmen compensation insurance policy coverage during 2021.

### Exclusion criteria

The study excluded the participants who could not be reached for an interview and were not capable of responding during the data collection period. The study participants’ records with missing gender and age were also excluded.

### Sample size and sampling techniques

The sample size was determined using single population proportion formula with the following assumptions: prevalence of occupational injury (p) = 78.3% [25], with a 5% margin of error at a 95% confidence interval and a design effect of 1.5, the calculated sample size yielded 1136 respondents. The study used a stratified sampling technique, expecting that workers in different work sections would have different levels of work-related injuries. The calculated sample size was proportionally allocated to each industry based on the number of injured cases. Data from administrative injuries records determined the total number of injuries during the reference period (January 1st to December 30th, 2021).

### Source of data for estimating the economic costs of occupation-related injuries

We used data from multiple sources to reduce the study’s reliance on workers’ compensation data alone for occupational injury. Finally, similar data were combined and complemented for further analysis. The following were some of the data sources used in the present study:

### Data from secondary sources

We used data records from the Bureau of Labor and Social Affairs to get all the injured cases in the reference period (2021). The study included variables derived from insurance companies, such as the name of the industry, premium payment, the number of injured workers (both non-fatal and fatal), the amount of compensation received, and the day of work missed. The study interviewed security officers, account department officers, occupational health and safety officers, and production managers. We incorporated the data obtained via a questionnaire with administrative records due to fear of high accident under-reporting.

### Data from manufacturing industries

The study gathered data on sick leave, the number of injured workers, the number of deaths, the total number of days lost, sick leave pay, and insurance premiums from manufacturing industries by using face-to-face interviews administered semi-structured questionnaire. The safety officers, plant managers, and industry insurance dealers collected additional data.

### Data from injured workers

The study collected the primary data from injured workers who have missed at least one working day to identify factors associated with the total cost variability after written consent was obtained. Workers meeting eligibility criteria were identified from administrative claims data by workers’ compensation a regulatory authority, which was triangulated with the self-reported data. Workers who do not opt out are contacted via telephone, and informed consent is sought. We used an interviewer-administered, semi-structured questionnaire adopted from International Labor Organization (ILO) injury statistics [26] with certain modification. To reduce the fear of COVID-19 infection, the data collectors strictly followed the recommended the standard protocol for COVID-19 prevention during the interview. Furthermore, the mask was distributed to all study participants and data collectors, and its use during the interview was strictly advised. Also, the data collectors and interviewees kept a two-meter physical distance. The interview was conducted separately in an adequate and well-ventilated room.

### Techniques and approaches to cost estimation

#### Estimation of direct cost of occupation-related injury

The study used insurance agency data to estimate the direct costs of injury through the top-down approach (allocating portions of a known total expenditure to each of several injury categories). The median direct expenses for healthcare and compensation were determined separately for each disability category and multiplied by the overall number of injuries. The study adjusted cost currency with international cash, the United States dollar (USD). For 2021, according to the commercial bank of Ethiopia, the average annual exchange rate between the USD and the Ethiopian Birr (ETB) was USD 1.0 = ETB 44.32 ETB [27].

#### Estimation of the indirect cost of occupation-related injury

To estimate the indirect costs of injuries sustained at work, we employed the friction cost estimation method. Using a multiplier, the present study accounted for employer productivity losses in absenteeism and presenteeism.

### Absenteeism cost estimation method

The study estimated absenteeism lost productivity due to the worker’s absence from work to quantify costs for employers. It was multiplied by the total number of absences from sick days by the median daily wage and the fractional value of the multiplier of absences from lost productivity. The median multiplier is 1.28, suggesting that the employer’s missing work cost is higher than the salary. The cost of absenteeism was calculated giving the following formula [28]:

1 $$\left[\mathrm{Absenteeism}\;\mathrm{costs}\;(\mathrm{AC})\right]\;=\left[(\mathrm{MLW}\;\times\;\mathrm{NIE}\;\times\;\mathrm{MDWE})\;\times\;1.28\right]$$

Where:

MLW = the median of lost workday’s due to absenteeism for the defined period,

NIE = the total number of injured employees,

MDWE = the median daily wage of the employees,

The median multiplier supports the view that the cost to the firm of missed work is often greater than the wage =1.28.

### Presenteeism cost estimation method

The researcher estimated presenteeism lost the cost of productivity due to the worker’s reduced job output. The cost of presenteeism was evaluated according to the following formula [29, 30]:

2 $$\left[Presenteeism\;\cos ts\;(PC)\right]\;=\;\left[(\mathrm{MLW}\;\times\;\mathrm{NIE}\;\times\;\mathrm{MDWE})\;\times\;1.5\right]$$

Where:

MLW = the median of lost workday’s due to presenteeism (the problem of workers’ being on the job but, because of illness or other medical conditions, not fully functioning),

NIE = the total number of injured employees,

MDWE = the median daily wage of the employees,

Presenteeism multiplier was used =1.5.

### Operational definition

**Absenteeism**: refers to the productivity lost when someone is absent for at least one day from the workplace because of an injury or illness for which the employee is accountable.

**Disability measurement:** temporary disability, permanent partial disability, permanent total.

disability and death [31, 32].

**Total cost:** referred to the sum of the direct and indirect costs of occupation-related injuries.

**Direct cost:** referred to expenditures associated with the usage of medical facilities and reimbursement (repayment) for payments made by organizations and insurance providers.

**Indirect costs:** referred to losses in production due to absence from work.

**Friction cost:** refers to the approach that measures the indirect cost of injury by estimating the cost of replacing those killed or temporarily or permanently disabled with other existing workers during the friction period.

**Occupational injury**: referred to any personal injury such as a cut, fracture, sprain, and so forth those results from a work-related event resulting in an absence from work of at least one day.

**Perspective (the level of analysis):** the point of view from which an analysis was conducted.

**Presenteeism:** reduced productivity, the performance of employees who work while they are sick or injured, or the practice of coming to work despite illness, injury, anxiety, etc., often resulting in reduced productivity.

**Unsafe Act:** Performance of a task or other activity conducted in a manner that may threaten the health and safety of workers. It includes improper use of PPE, operating equipment at an unsafe speed, bypassing or removing safety devices, using defective equipment, using tools other than their intended purpose, working in hazardous locations without adequate protection or warning, and improper equipment repair. We asked about 15 questions, and respondents’ yes response scores were 1, and no scores were 0. Then, the proportions of unsafe acts were computed by pooling multiple responses.

### Data quality assurance

Trained data collectors (*n* = 6) with a degree in occupational health and safety gathered the required data. The principal investigator trained data collectors and supervisors for two days. The training focused on the data collection tools, the study procedures, and research ethics. The questionnaires were translated into the Amharic language by an experienced translator and back-translated into English by an independent translator for consistency. A pretest was done on 10% of the sample size outside of the study area before data collection. The principal investigator checked the collected data for completeness and consistency before further analysis. The principal investigator closely monitored the field-level data collection process daily. The investigator approached the data collectors when errors were noticed, correcting the field level.

### Data processing and analysis

The study cleaned up the data, did an inspection of distributions, and cleaned contingency for accuracy. We executed case sorting to find the missing variables. Data were coded and entered into EPI-INFO version 7 and exported to the STATA 14 software [33] for further analysis. The data analysis was conducted in a step-wise manner, in which first the characteristics of study participants were analyzed and described. The direct, indirect, and total costs of occupation-related injuries were analyzed. We converted all cost information to United States dollars (USD$). The study checked multi-collinearity using a correlation matrix at > 0.8, a variance inflation factor (VIF) > 10, and a tolerance of 0.1. It was tested for model fitness using the Hosmer and Lemeshow test at a *p*-value > 0.05.

Next, the cost of occupational injury data was checked for normality using plots (Q-Q plots and histograms) and the Kolmogorov-Smirnov test for normality (*P* > 0.05). The cost data was discovered to be right-skewed, and a log transformation was performed to confirm the normality of the skewed data. The study employed a generalized linear model (GLM) with a gamma family and log link function to identify predictors of total cost by considering the non-normal distribution of the total cost. Exponentiate coefficients (exp (b)) with a 95% confidence interval were used to express the direction and strength of the association. Variables with *p*-values of 0.05 at a 95% confidence level were considered statistically significant in the study.

## Results

### Socio-demographic and injury characteristics of participants

There were 1136 eligible participants in the present study, which resulted in a 100% response rate. During the reference period, 1200 injury data sets were collected from employee injury compensation claim records. It was found that 607 (50.6%) and 476 (39.7%) of the victims experienced permanent partial disability and temporary total disability, respectively (Table 1).

**Table 1:** Socio-demographic characteristics of injured workers in manufacturing industries in Ethiopia, 2021

Characteristics		Frequency	Per cent
Sex	Male	1175	97.9
	Female	25	2.1
Type of injury characteristics	Temporary disability	476	39.7
	Permanent partial disability	607	50.6
	Permanent total disability	70	5.8
	Death	47	3.9
Injured body parts	Hand and finger	720	60
	Leg and foot	2017	17.3
	Head, and neck	140	11.7
	Eye	48	4
	Ear	8	0.7
	Back and vertebra	8	0.7
	Chest and shoulder	8	0.7
	Teeth	32	2.7
	Multiple location	29	2.4
Cause of events	Slippery surface	258	21.5
	Contact with objects and equipment	123	10.3
	Working from height / fall	271	22.6
	Exposure to harmful substances	141	11.8
	Transportation accidents	63	5.3
	Fires and explosions	57	4.8
	Machine	52	4.3
	Miss handling	53	4.4
	Lifting heavy material	27	2.3
	Hand tools	52	4.3
	Sweaty palms	103	8.6

### Personal and clinical characteristics of participants

This study demonstrated that long-term absence from work and sleeping disturbances were experienced by 933 (82.13%) and 636 (55.99%) of the study participants. Besides, 740 (65.14%) of the study participants reported using personal protective equipment inconsistently (Table 2).

**Table 2:** Personal and clinical characteristics of the study participants in manufacturing industries in Ethiopia, 2022

Characteristics		Frequency	Per cent
Injury prevents from work	Yes	684	60.20
	No	452	39.80
Unsafe act of the workers	It is not a case	70	6.16
	Is a case	1066	93.84
Duration of absence from work	short –term absence	202	17.78
	long-term absence (> 5 days)	933	82.13
Having sleeping disorder	Yes	636	55.99
	No	500	44.01
Co-morbidity	Yes	636	55.99
	No	500	44.01
Personal protective equipment (PPE) utilization	Consistent use	396	34.86
	Inconsistent use	740	65.14
Reasons for not using PPE	Factory not provide it	540	16.90
	Not comfortable to use/lack fitness	1060	33.20
	Lack of knowledge on how to use it	229	7.20
	Decrease work performance	1361	42.70
Severity (type) of injury	Minor injury	452	39.79
	Severe injury	684	60.21
Timing of injury happens	Morning	414	36.44
	Afternoon	235	20.69
	Night	487	42.87

### Loss of workday (absence)

The present study illustrated that the total number of days injured workers were away from work was 29,891 workdays during the reference period. In this regard, the median number of days away from work (absence workday) was 21.48 days.

Regarding the proportions of the ranges of workdays lost, about 894 (74.5%) and 258 (21.5%) victims were absent from work due to occupation-related injuries for 5–30 days and over 30 days, respectively. Lastly, about 48 (4%) of the injured cases were absent from work during the reference period.

### Economic costs of occupation-related injuries

#### Direct cost

The present study used the median cost of injured cases (medical costs and disability compensation payments) and multiplied them by the total number of injuries to estimate the total direct costs. Accordingly, the total direct cost was 7,711,584 Ethiopian Birr (173,997.83 USD). The median direct costs (per victim) were 6426.32 Birr (144.99 USD). Besides, compensation payments amounted to 2,516,754.72 Ethiopian birrs (56,785.98 USD) (Table 3).

**Table 3:** Medical and compensation claim payment of occupation-related injuries in manufacturing industries in Ethiopia, 2021 (*n* = 1200)

S.n	Cost categories		estimated cost in Ethiopian birr (united state dollars,$)	Median ($) of Cost
**1.**	Medical cost		962,102.42 (21,708.08$)	3000 (67.68$)
**2.**	Compensation cost	Accident classification		
		Temporary total disability	479,188.56 (10,812.01$)	3426.32 ($)
		Permanent partial disability	506,188.34 (11,421.21$)	
		Permanent total disability	752,189.02 (16,971.77$)	
		Fatal-injury	779,188.8 (17,580.97$)	
		Total compensation cost	2,516,754.72 (56,785.98)	
3.	Amount of premium pay		15,135,322.68 (341,500.96$)	
4.	Total direct cost		7,711,584 (173,997.83$)	6426.32 (144.99$)

#### Indirect costs

In the present study, indirect costs incurred were estimated through lost productivity that results from injury-related absences. To estimate total production costs, the median wage of the injured employee was taken to calculate the median daily salary. The median daily wage of the employees was 207.6 Ethiopian Birr (60.7 SD). Also, the median workday lost was 21.48 days, resulting in a total indirect cost of 14,876,051.32 Ethiopian birrs (303,592.88 dollars). Additionally, the presenteeism cost constitutes 8,026,646.40 Ethiopian Birrs (163,809.11 dollars) of the whole production cost (Table 4).

**Table 4:** The indirect cost of occupation-related injuries by cost components in manufacturing industries in Ethiopia, 2021 (*n* = 1200)

S.n	Costs component	Estimated costs in birrs (USD $)
1.	Absenteeism, estimated cost	6,849,404.92 (139,783.77$)
2.	Presenteeism, estimated cost	8,026,646.40 (163,809.11$)
3.	Total indirect cost	14,876,051.32 (303,592.88$)
4.	Average cost (total costs divided by the no of injured cases)	12,396.709 (252.99$)

### Total costs of occupation-related injury

In this study, the total costs were determined by summing the direct and indirect costs of occupation-related injuries. The total cost of occupation-related injury to the employers, according to the current study, was 22,587,635.32 Ethiopian birr (537,800.84 dollars). The indirect cost accounted for 65.86% of the whole expense, and the direct cost incurred was 34.14%. The compensation expenses accounted for approximately 2,516,754.72 Ethiopian birrs (56,785.98 $) of the total costs, while presenteeism costs accounted for 8,026,646.40 (163,809.11 $) (Table 5).

**Table 5:** Total costs of occupation-related injuries by the reference period in manufacturing industries in Ethiopia, 2021 (*n* = 1200)

Cost category	Cost estimation period, 2021	
	Cost in Ethiopian Birr (USD$)	% of the total cost of injury
Direct costs	7,711,584 (173,997.83$)	34.14
Indirect costs	14,876,051.32 (303,592.88$)	65.86
Total costs	22,587,635.32 (537,800.84$)	100

### Predictors of total costs of occupation-related injuries

The study was fitted with a generalized linear model (GLM) to identify the potential predictors that influenced the total cost variability. Long-term absence from work (exp (b) = 0.85), severity (types) of injury (exp (b) = 1.11), unsafe acts of workers (exp (b) = 1.44), sleeping disturbance (exp (b) = 0.90), and co-morbidity (exp (b) = 0.85) were statistically significantly associated with total cost variations in the fully adjusted model (Table 6).

**Table 6:** Generalized linear model analysis to identify the predictors of the total cost of occupation-related injuries in Ethiopia, 2022 (*n* = 1136)

Total costs of occupation-related injuries		Exp (b) the corresponding Adjusted odds ratios (AOR)	Std.error	(95% Conf. Interval)	
				Lower	Upper
Duration of absence from work	Long-term absence (> 5 working days)	0.85	.004	0.80	0.90
Severity (types) of injury	Yes (it was a case)	1.11	0.03	1.02	1.21
Unsafe act of the workers	Yes (it was a case)	1.44	0.01	1.20	1.72
Having sleeping disorder	Yes (it was a case)	0.90	0.004	0.82	0.98
Having co-morbid illness	Yes (it was a case)	0.85	0.004	0.78	0.92
PPE utilization	Inconsistent use	0.96	0.004	0.89	1.05

### Interpretations of generalized linear model outputs

The long-term absence of employees from work due to work-related injuries was statistically significantly associated with a variation in the total cost borne by employers. So, for each one-unit increase in the long-term absence total score, the odds of being in total cost variations paid by employers were increased by 15% (Exp (b) = 0.85 (95% CI = [0.80, 0.90]) after controlling for all other covariates in the model (Table 6).

Furthermore, the severity (types) of injury was statistically related to the total cost of occupational-related injury variation from the perspective of employers. In this manner, for every 1 point increase in the severity of injury total score, the odds of being in total cost variation were elevated by 11% (Exp (b) = 1.11, 95% CI = [1.02, 1.21]) after controlling other factors in the model (Table 6).

Similarly, an unsafe act by the workers was associated with a substantially heightened total cost of occupation-related injury variation in employers’ views. In this regard, for each one-unit increase in an unsafe act’s total score, the odds of being in total cost variations are raised by 44% (Exp (b) =1.44, 95% CI = [1.20, 1.72]) after adjusting other covariates in the model (Table 6).

Additionally, having sleep disturbances was associated with a variation in the total cost of work-related injuries, depending on the viewpoint of the employer. In this way, for each one-unit increase in the sleeping disorders total score, the odds of being in total cost variations increased by 10% (Exp (b) = 0.90, 95% CI = [0.82, 0.98]) after controlling other covariates in the model (Table 6). Likewise, for each one-unit increase in co-morbid illness, the total score, the odds of being in total cost variations were elevated by 15% (Exp (b) = 0.85, 95% CI = [0.78, 0.92]) after controlling other covariates in the model (Table 6).

## Discussions

The present study’s objective was to estimate the costs of occupation-related injuries and their predictors from the perspective of the employer in Ethiopia. The data-driven evidence on the economic cost of occupational injuries should provide relevant stakeholders with a better understanding. This insight assists them in making improvements to the working conditions of industrial workers and can positively impact policymakers’ decisions, including improving organizational productivity.

Moreover, comparing the costs of work-related injuries is challenging due to the dissimilarity in currency and exchange rates across countries. However, these differences have much in common, like determining eligibility for claims and paying for medical care and other benefits. At times, it was believed that taking into account the proportional contributions of the various costs of injury components may offer a better comparison method. The previous research reported its results without taking into account the variations in currency exchange rates between the various countries. To the authors’ scope of searching, we lacked specific information about how earlier research had presented their results in such circumstances. On the other hand, countries usually peg their currencies to maintain stability for investors, who don’t want to worry about fluctuations in the currency’s value. The present study found that the total cost of occupation-related damage incurred by the employers was incredibly higher than what had been previously estimated in Ghana [34], America [8],Croatia [35], India [36],Malaysia [37], Netherland [38], and Thailand [9]. This substantial amount of cost variation was explained by cost estimates, the particular costing approaches and the perspectives used the data collection methods, and the sizes or composition of industries. The structure of occupational health and safety services may not yet be strong enough to address the rising demands for workers’ health in the context of industrialization, which is another potential explanation for this disparity. We suggested that expenses due to work-related injuries are very high due to the lack of simple preventive measures in the present study location.

Conversely, our cost estimates are considerably lower when compared to a similar study done in South Africa [39], Poland [40], other parts of the United States [41], Australia [42], Finland [43], and Mexico [44]. The estimated disparity is explained by the differences in the sources of data used, the range of cost components included, and the reference period considered. Besides, the cost computations in our study were limited to medical care, compensation, and productivity loss. Also, our cost estimates were limited to the employer side in one calendar year. The previous literature has estimated the cost of work-related injuries from employers, employees, and social perspectives, which might heighten the discrepancy.

Furthermore, a substantial literature has shown that occupation-related fatality is becoming the overwhelming issue accountable for huge economic losses. For instance, the pooled prevalence of occupational injury in Ethiopia was 44.66% [45], in Ghana 57.91% [46], and 57% in the Africa region [47]. These imply that the burdens of work-related injury are elevating; the economic burdens associated with work-related injury will be expected to be high. Additionally, a researcher with different costing approaches and perspectives reported that the total cost of a workplace injury is often underestimated because some cost components are challenging to quantify, and there is an underreporting of uninsured injuries. Also, the indirect costs could be four times higher than the direct costs [48]. Similar to the above study, the present study found that the indirect cost comprised the largest component of the total cost and was much greater than the direct cost incurred. Compared to the direct costs, there are many variations in the proportion of the expenses, but usually, the proportion of indirect costs is potentially more costly than direct costs. This is consistent with evidence from five European Union countries [49], Canada [50], Turkey [51], Malaysia [52], and German [53]. Evidence showed that direct cost is usually something that can be known at the time of the accident. In contrast, indirect cost needs to be quantified after the accident [54, 55].

Moreover, the present study revealed that the total indirect cost incurred was much higher than the finding from Mexico [44, 56], Ghana [57], and Europe [49]. The possible reason for this discrepancy might be due to the cost categories considered and the costing approach used. However, our study estimated the indirect cost only from productivity losses, and other indirect cost components were not included. In our research, presenteeism cost was the largest component of indirect costs than absenteeism borne by the employers. These increased costs of productivity losses imply that the injured worker was present at work but contributed a sub-normal individual output due to incomplete recovery, which could be leading to lower productivity. The other literature supported our findings [58, 59]. Additionally, the decrease in productivity translates to the inability to perform routine tasks [60]. Also, reduced on-the-job productivity due to health issues affecting the overall performance of companies with negative economic implications to be associated with presenteeism [59, 61].

Similarly, the compensation cost consists of the most significant direct cost component compared to direct medical costs. On the other hand, the direct medical price is much higher than the finding in Turkey [62]. The more accidents organizations have, the more expensive the coverage gets. Workers’ compensation payments are determined by the cost of worker injuries that could be elevated then next year’s premiums more likely. Also, it might be related to the nature and the size of the industries.

Furthermore, knowing the factors influencing cost variability allows the employers and policymakers to identify the area of focus for better decision-making. Our generalized linear model indicates that long-term absence from work considerably increases the total cost variation, which is in line with previous studies conducted in different countries [63, 64]. Employers’ economic expenses are quite significant when people are absent from their occupations for an extended period of time, which affects productivity. Employers also have a legal obligation to provide compensation and cover the costs of a long absence, which adds to the cost disparity. Long-term sick leave also raises the likelihood of chronic impairment, putting employers under additional hardship.

In the context of our search, no literature has examined the influence of unsafe actions of workers on employers’ ‘side total cost variability. Yet, our findings revealed that an unsafe act by the workers was a significant predictor of the total costs. This is mainly associated with unsafe acts of workers that could lead to severe injury or death and which raise the economic burdens of employers as unsafe behaviors of workers are essential contributors to occupational injuries. Related studies found that 88% of workplace incidents in the industry were caused by unsafe behaviors and 10% by unsafe physical conditions [65–67].

Likewise, our finding documented that having co-morbid illness causes raised employers’ expenditures. This could be because when individual workers are absent from work due to poor progress health, the financial expenses born by employers are expected to be elevated. Also, co-morbid health problems may reduce the workers’ work performance and efficiency and potentially impact the workers’ skills. Thus, our finding was supported by evidence from Florida [68], and England [69]. Similarly, our results indicated that sleep disturbance significantly influenced the total costs, which ultimately can lead to a significant economic weight on employers. This means that as sleep disturbance is responsible for a significant driver of fatigue, the cost of work-related injury was hugely higher among employees presented with sleeping disturbance. The other possible explanation for why sleeping disorders could be potential predictors of the total cost is that workers are more prone to slow thinking, disorientation, and mistakes and loss of work function the longer they go without sleep. As a result, the risk of harm and death to workers and anyone nearby rises, which is expected to raise the cost of a work-related injury. Numerous well-known industrial tragedies have been linked to fatigue as one of the primary contributing factors. The present finding is supported by the result from Australia [70], Iran [71], Switzerland [72], America Insomnia Survey [73]*,* and Korea [74]. However, we suggest an in-depth estimation of the real cost of sleeping disorders; such a study would have paramount importance.

Besides, our results demonstrated that the severity of the injury was suggested as the predictor of the variation of the total cost. This means that as the injured workers didn’t recover in the shortest period due to the severity of injured body parts and associated complications, the prices of injury from employers’ views were considerably large. One study has also revealed similar results as we did [75]. The literature showed that most common costs are sickness absenteeism, health care, individual productivity losses or presenteeism, insurance and pension costs, and indirect costs, such as hiring a replacement and paying for overtime [76, 77].

### Public health implication

The present study findings increase our knowledge of the economic consequences of injury and provide vital information for further economic analyses. Knowledge of occupation-related injury costs can help decision-makers decide which occupational health problems need to be addressed first and efficiently allocate health and safety resources. Also, it is implied that these substantial economic costs of injuries reflect negatively on productivity due to increased absenteeism, decreased production, and higher insurance and workers’ compensation premiums. Additionally, for specific stakeholders, such as the Ministry of Labour and Social Affairs and the Ministry of Health, the cost of an occupation-related injury study can show the injury’s financial impact on industrial productivity and motivate them to undertake injury prevention initiatives. Policymakers can better prioritize occupational health and safety measures with the aid of estimates of the financial cost of work-related injuries.

## Strength and limitations of the study

One of the study’s strengths was selecting the most significant manufacturing industries to make the results representative of most of Ethiopia’s manufacturing industries. We also applied a generalized linear model that is more robust to the non-normal distribution of estimate. The other strength of our study was that we incorporated a top-down approach and a friction cost estimation method. The findings from our research have their own set of limitations. One limitation is that we didn’t consider occupational diseases. The other limitation is that the costs of occupational injury from workers’ perspectives and societal levels were not considered. In addition, all components of indirect costs and direct non-medical costs incurred by employers were not covered within the scope of the current study. Also, the cross-sectional nature of the study limits the external validity of the study. Finally, we are unable to compare time and industries in Ethiopia due to a lack of comparable research.

## Conclusions

Occupation-related injuries exert a substantive impact (cost) on the employers and have severe economic implications. This study adds to the growing body of evidence that improving the working conditions of industrial workers could positively affect the economic burden of work-related injuries. Moreover, the long-term absence from work, unsafe acts of the workers, co-morbidity, sleeping disorders, and severity of injury were influential factors that elevated the total cost variation.

Consequently, we recommend that the modifiable factors identified in the present study be the areas of intervention to reduce the cost of occupational injury. Also, the employers and all parties should play a leading role in the *anticipation of problems through risk assessment and* reducing claim frequency to reduce workers’ compensation board premiums. Besides, the employers should enforce the implementation of occupational health and safety measures in their firms. Additionally, in order to dramatically reduce the costs of occupational injuries, employers should take aggressive steps to reduce the number of work-related injuries and improve the dissemination of safety information to employees. To obtain a complete picture of the burden, the researcher should conduct a follow-up study to determine whether the economic burden of occupational injury will increase from both the employee and societal perspectives.

## Data Availability

Data reported in this manuscript are available from the corresponding author on reasonable request.
